# Supplementary material for: Patterns of Intron Gain and Loss in Fungi
Source: PLoS Biol. 2004 Nov 30;2(12):e422. doi: 10.1371/journal.pbio.0020422 (PMC532390; doi:10.1371/journal.pbio.0020422)
Supplement: Table S1 — Also available at http://genes.mit.edu/NielsenEtAl/. (4.3 MB ZIP). [file pbio.0020422.st001.zip › NielsenEtAl/html/577.html]

AN4890.1.NCU09432.1.MG00926.1.FG00343.1


```
 CLUSTAL W (1.82) Multiple Sequence Alignments - Introns Inserted


Sequence 1: NCU09432.1	999 aa
Sequence 2: MG00926.1	1051 aa
Sequence 3: FG00343.1	983 aa
Sequence 4: AN4890.1	1329 aa
Alignment Length: 1346 aa
Number Identitical Residues: 350 aa
Alignment Score (without introns) 20761


MG00926.1 	------------------------------------------------------------
NCU09432.1	------------------------------------------------------------
FG00343.1 	------------------------------------------------------------
AN4890.1  	MFPPLPAPSDPEKEVSDKLVSILRRHFLVPIHVDRIWPLAGHLHSFYLVQLSNETHLLLK
          	  .. .:.:..... :..  :        .   .   . :.   :    . :..:    .

MG00926.1 	------------------------------------------------------------
NCU09432.1	------------------------------------------------------------
FG00343.1 	------------------------------------------------------------
AN4890.1  	CSPKPTTPLIRREHIMLDTEARALSLLSHMPCIPQLLHYDPLGDLLGPSFLLRHYIPGTS
          	.:...::.    .    .:.: : :  :  .. ..    .. ..  ..:       ..::

MG00926.1 	------------------------------------------------------------
NCU09432.1	------------------------------------------------------------
FG00343.1 	------------------------------------------------------------
AN4890.1  	LDKIDSQLSIQERDSIERSLGLLAKRIAQHTSESFGSLGQVASAGGTKSWREGFLILFEG
          	 .. .:. : .. .: . : .  :.  :. ::.: .: .. :::..:.:  ..     ..

MG00926.1 	------------------------------------------------------------
NCU09432.1	------------------------------------------------------------
FG00343.1 	------------------------------------------------------------
AN4890.1  	LLRNAEDVFINLPYGEIRYQVHRASPALDEILTPRLVVIDFGQPSQVLVNESKRLCGISS
          	   .:..   . . ..   .   ::.: ..  :.     . ...:.   ..:.  .. ::

MG00926.1 	---------------------------MEATRQDVGGGRVTV~VKNRLLTPAAVALQLQR
NCU09432.1	------------------------------------------~-----------------
FG00343.1 	------------------------------------------~-----------------
AN4890.1  	FRTAVWGDVFMAKIFENPSPSVIDGFGSCYAKTQTGGTRQLL2YSCYRAVNDVVLQYYRK
          	  ::  ..   :.  ...:.:  .. .   :  . ..       .               

MG00926.1 	WDGGRQVGEVTCAAPHHCKLAV1V~QLSFSLVTS~AMASFLRSRQAGMNVDLSVKIEPQH
NCU09432.1	----------------------~-~---------~-MASFIRAKQAGVQTDLSQNIQPMH
FG00343.1 	----------------------~-~---------~-MAAFLRSKQAGMQNDLSASIRPEL
AN4890.1  	SRDTAAEIKARRELMTALAKMA~A0RAAVAYIAF0EMAHFLRGKQAGIQKDFSGSLAPEL
          	  .     .                  : :  :   ** *:*.:***:: *:* .: *  

MG00926.1 	FSPAE~QARYGINSQIS2CVAYDPVQSLIAVGTNESKFGNGKIYIFGQSRVQKCISPNKQ
NCU09432.1	FMPDE~QARYGINSQIS~CLAYDPVQSLLAVGTNESKFGPGKIYVFGQRRVHKFFCPPRP
FG00343.1 	FMPDD~QARYGINSQIS2CYAYDPVQSLLAIGTGESKFGPGKVYIFGQRRVHKILEPPRS
AN4890.1  	FDIDD0LLRCGINSQTS~ALAYDPVQSLLAVGTSESQFGHGQVYVFGQGRVSVIFSLPRK
          	*   :   * ***** * . ********:*:**.**:** *::*:*** **   :   : 

MG00926.1 	ASIRFLHFVADRLVSLDSRSELSIWDLDTAQRLGGYGNAGAVACMVTDPMLDWVLMGTTS
NCU09432.1	SSIRELRFVANRLISLDSKNELAIWDLDTGNQVAKYVYSG-VVCLVTDPMLDWAFVGLQS
FG00343.1 	TSFQIVQFSSNRLVTVDAKNELGIWDIDTGERVAAMVVAGQVVSLVTDPMLDWAFIGLYN
AN4890.1  	ASAKFLQFCGDKLISVDSKSEISVFSLETKKLLVSYAPPSIASALLSDPSLDYAFIGLQN
          	:* : ::* .::*:::*::.*:.::.::* : :     .. . .:::** **:.::*  .

MG00926.1 	G~DVFAFDLDRSRLS-NFRLPNFWKQRDPRVRAPNMVSMQLHPRDIGKLLIAYNSGAVIY
NCU09432.1	G~DIAAYDLDRERPS-TFRIPNFWRERDPNPTAGRLVSMQLHPRDIGKILIAYTNGAVIY
FG00343.1 	G~DIMAFDLDRGNLARAFRLPNFWKERNPGAATSNLITMSLHPRDVGKLLLGYTHGAVVY
AN4890.1  	G1DVIAYDLDRETVT-PYKISNLWAQRNPRARICPVISLAFSPRDIGKILIGYPDGAVTF
          	* *: *:****   :  :::.*:* :*:*      :::: : ***:**:*:.*  *** :

MG00926.1 	SFKQNQPTRFFEYVLQPGAPGGTGDK-VNTVRTPRVIQAVWHPTGTFVLTAHDDGSLVFW
NCU09432.1	SFKQNIVQKYFEYELPPDAPGGDGHG-IHMARKPKITHAVWHPTGTFILTAHEDGSLAIW
FG00343.1 	SFKQAKAVNYYEYVVPAGAPGGNGVA-VETVRKPRLTHALWHPSGTFVLTVHDDGSLVFW
AN4890.1  	SFKQNVAQHYFEYEVPPGALGGNGIAPYRDIRKPKLTKALWHPNGIFILTVHDDNSLVFW
          	****    .::** : ..* ** *  . .  *.*:: :*:***.* *:**.*:*.**.:*

MG00926.1 	DAKDGRMIAARSLSEINVDQPTAEPPRP-------VLLEPFTHIAWCCKNDPDDTGLLIA
NCU09432.1	DTKEGRLVTARSLYRTRVNEPTSNPPRP-------MRLAPFVRISWCCQTDPDNTGLLIA
FG00343.1 	DPKAEKVVMARTLTDFDVDQPSSATSVP-------ALSEPITKIAWCCKENCDDSGLLIA
AN4890.1  	DTKDGRKLLARSITTPNIDQPGVGPRGPAAPESAISLKDPITDVAWCVKANGDDSGLLIA
          	*.*  : : **::    :::*   .  *::..::     *:. ::** : : *::*****

MG00926.1 	GGLPVGS-TEKGLTFLELGPTPIYATSSWEVLSNHFKG-KRQHLLRAPLGADITEFYLLP
NCU09432.1	GGHNIDDPTASNLTFLELGPTPIYATSSWEVLAAHFDG-KRSLPISTPPGASVVNYVLIP
FG00343.1 	GGQRMDA-SPKGLTFVDLGQTPVYATSTWQILATYCRG-KRQSLLQLPPGAQAINFLLIP
AN4890.1  	GGKPKDE-RCKNLTFIDLGPSPNYQTSSWAMITTYLENPKTIAEISIPPGAEVVRFCLIP
          	**   .    ..***::** :* * **:* ::: :  ..*    :  * **.  .: *:*

MG00926.1 	RSSPHFAGAQDPIAVIALLNSGEMITMSFPSGYPISPTNQLHPSLTFVHPFVMKVAVTTV
NCU09432.1	RSSPYFNGAQDPIAVLVQLSSGELITLTFPSGFPISPTNMLHPSLSFVHPFVQTVAVSTV
FG00343.1 	RFSPHFGGAQDPIAVLALLSSGELITLSFPSGYPISPTNQLHPSVSFVHPFVTKVNVSAL
AN4890.1  	RNSPYYGGAHDPIAILALLTSGELITLSFPSGHPIPPTNMLPPSLTLIHPFVNKITLTPV
          	* **:: **:****::. *.***:**::****.**.*** * **::::**** .: ::.:

MG00926.1 	SRERWLGMIEVRNQGEPLLKGGALARRLRRRHDSRNIIQVAHADGTVRLWDVGHGDDIEN
NCU09432.1	PRARWLGMAEKRSLGEQILRGG-VEYSHRTRKEQRNIVQVLHADNIIRLWDVGRGDEIEN
FG00343.1 	ERPRWLSMFEKRNQGEPLLKGGAEAPRPRKRFEERTIIQAAHADSTIRIWDSGHADEIEN
AN4890.1  	DRADWLGLKERRSQGPKFILGGAEERKQLKRLESRNIITAAHADGTVRIWDVGHDDEIEN
          	 *  **.: * *. *  :: **:       * :.*.*: . ***. :*:** *: *:***

MG00926.1 	AGMLQVDVARSTGRYED-LEITALSMASHTGEFAAGTKTGEVAIYRWGTNKHFQHDSETD
NCU09432.1	AMQCQVDVARSLNRFEGGVETTAMHMAQETGEFAAGTKTGEVIVWRWGINKNFGKEAPRA
FG00343.1 	GTQLQVDVARALDRYED-VEITCMNMATSTGEFVVGTRTGEAIVYRWGQNRFFGKDSMPE
AN4890.1  	SELIQVDLARALGRVGV-VDVTEMSLGGSSGELSVGLRSGEVAIFRWGSNQNFGLEQPPG
          	.   ***:**: .*    :: * : :.  :**: .* ::**. ::*** *: *  :    

MG00926.1 	IKSNPGGLTDISIRSEPSLREGLQPLILYEMMQGPITALQVSNVGFVAVGSENGFFSIID
NCU09432.1	EECERGGLMDISSRAEPSLKEGLQPFVMHNMAQGRVTVVAVSDVGFVAAGSEGGQLSIID
FG00343.1 	LDPNPKGLTDISSRAEPGLKEGLQPLVLYEMMMGPITAVQVSNVGFVAVGSELGFLTLID
AN4890.1  	TNNGPGELTDITHRTDPSLKHGLLPLTLLSMQQGPVTALKHSPIGFVAAGFEGGSIAIID
          	 .     * **: *::*.*:.** *: : .*  * :*.:  * :****.* * * :::**

MG00926.1 	LRGPAIIMQASIAEFTKQEKRSSFLRGHSSGSSGK-DWPVVIEFGVMTLEDDN~YSSIAC
NCU09432.1	LRGPKVIYHGSVTEWSKGEKRGLFKHSSSSSHGGKPEWPTVIEFGVMTLEGEN~YSSICC
FG00343.1 	LRGPSIIFQAPMTDFAKQDKRASFLKGHSSSEPPK-EWPVVIEFSVMTLDEDK~YSSICC
AN4890.1  	LRGPAIIHTANISELSKQNKRSSFLKTRSSSETIP-EVPTAIEFGILTLEGEE1FSSICC
          	**** :*  . ::: :* :**. * :  **.     : *..***.::**: :: :***.*

MG00926.1 	FVGTNQGKVATFKLIPSGGG-YSAKLAGVVNCGDQIISISPIMTETGAPAAATGEIVAGL
NCU09432.1	FVGTNMGRVATFKLLLSGQG-YSVQLAGVANMDDKVVAICPIEVQTGREAGATGPIVAGL
FG00343.1 	FVGTNLGKVITLKILPGQDGTYTAQPAGVVAFDGKVISLSPIEADTGKPALATGPIVAGL
AN4890.1  	FVGTNRGNLATFKILPSSGGAFVASFAGAVALEDKIINIIPINADDGSLALATGKAFGGL
          	***** *.: *:*:: .  *:: .. **..   .::: : ** .: *  * ***  ..**

MG00926.1 	REGKQVNGTLVVA1TQTEIRIFKPATAKGASKSFDN-VFCDAAAVT----EFELHGHAIV
NCU09432.1	REGKQVDGVLVAV1TQTESKIFRPPHARGASKSFDSGILCDAAAVTPADTSMSSHGYALV
FG00343.1 	REGRQVNGVLVAV1TQTEIRIFKPANSKGASKEFDD-ILCDAACVA----ETELQGFAVV
AN4890.1  	RNGTRVHGAVVAV~TVNSCRIFKPATSKGAHRSWDD-YLCDSASVVN----LPGRGCSLV
          	*:* :*.*.:*.. * .. :**:*. ::** :.:*.  :**:*.*.        :* ::*

MG00926.1 	GVFGDCTARAFSIPGLKEISKASLNGTMDPGRSGGSLVTPTGDIFCWAGPSELVAVPVWG
NCU09432.1	AVFNDRTLRSYTLPGLREIAKHPLP-MLDPLRTISTVISKTGDVLGFTGPSEITVLPVWG
FG00343.1 	GLFGDRTARAFSIPALKDIGKADLP-MIDVTRTTNSTVTQTGDIFAWSGPSELAVIHVWG
AN4890.1  	GLFGDGNARAFSIPGLKEIGCAKIGHMADMSRLSQSTVSPNGSVLFWTSPSEIGLINVWG
          	.:*.* . *::::*.*::*.   :    *  *   : :: .*.:: ::.***:  : ***

MG00926.1 	G-GTGV~SNSDDTMINPAMKVPPRPTISNMQWISGTQHVSPTDLDELI~GGPDRPPSKRM
NCU09432.1	GSGRAL~ENTLDTMINMAQELPPRPTISNVQWLSGTQYISPTDLDLLI~GGPDRPPSKRM
FG00343.1 	A-GKGP~QQSPDTMINPKLECPPRPTISNMQWISGSQHITPADLDLLV~GGPDRPPSKRM
AN4890.1  	A-GIEL2RPSEDQLFNPQAVSPPRPTITNLQWISGTQYISTADMDILI1GGPDRPPSKRM
          	. *      : * ::*     ******:*:**:**:*:::.:*:* *: ***********

MG00926.1 	IAAAAEEQRLA-----RGGGVSSSSTAMRAG--ATQEGWGEYLTRQLNERTEKLNIVNDS
NCU09432.1	MAATDAERNGAGVYNPAAAGAAGSSTARLQGQVKETEGWGDYLTRQLNERTEKLNIMGDS
FG00343.1 	MDEAAAEQRAAR---AGEPGPAAGSS--------SQEGWGDYLTRQLNERTEKLNMMGDN
AN4890.1  	IEQMRLEEQER-----RQAAREGRSTPARQN--TNDEGYFAYMQRQVQERTEKLSFAGDN
          	:     *..          .  . *:.   .     **:  *: **::******.: .*.

MG00926.1 	MDNLQTKSQGWADDVNKYVSKQKKNLVLGA~IKGKFS-
NCU09432.1	MDNLANTTSKWAEDVDGFVKKQKRDLFLGG~IKKSFF-
FG00343.1 	MENLQNNSAGWADDVNKYLNKQKKNVILGG~LKSKFF-
AN4890.1  	MDRLEETSSGWARDVNKYVQNQKKKAVLGV1LGSKFGL
          	*:.*  .:  ** **: ::.:**:. .**  :  .*
```
